# Long-term clinical outcome of lung radiofrequency ablation in patients with musculoskeletal sarcoma

**DOI:** 10.1007/s10147-026-02975-7

**Published:** 2026-02-04

**Authors:** Tomoki Nakamura, Masashi Fujimori, Takashi Yamanaka, Tomohito Hagi, Yumi Matsuyama, Masahiro Hasegawa

**Affiliations:** 1 Department of Orthopaedic Surgery, Mie University Graduate School of Medicine, 2-174 Edobashi, Tsu city, Mie 514-8507 Japan; 2 Department of Radiology, Mie University School of Medicine, Tsu city, Mie 514-8507 Japan

**Keywords:** Radiofrequency ablation, Lung metastasis, Soft-tissue sarcoma, Prognosis, Lung function

## Abstract

**Background:**

Long-term outcomes in patients with lung metastases from sarcomas remain unreported. We retrospectively evaluated the clinical utility of lung radiofrequency ablation (RFA) in 52 patients with musculoskeletal sarcoma-derived lung metastases.

**Patients:**

The study cohort included 29 men and 23 women with a mean age of 55 years at the time of the initial lung RFA, with a mean follow-up duration of 49.2 months. Complete treatment was defined as achieving a tumor-free status following the initial lung RFA. Cases failing to achieve this were classified as incomplete treatment.

**Results:**

At the final follow-up, 14 patients remained alive, while 38 had died from this disease. Multivariate analysis confirmed that complete ablation and longer disease-free interval were significant prognostic factors. The median survival time for the 27 patients with complete treatment was 96.7 months, compared with 13.1 months for the 25 patients with incomplete treatment. The 3- and 5-year survival rates after the initial RFA in the 27 patients with complete treatment were 55.3% and 51.4%, respectively, whereas the corresponding rates for the incomplete treatment group were 16% and 10.7%. Of the 27 patients who achieved complete treatment, 4 had no new lung metastases, whereas 23 developed new lung metastases and/or local relapse. Local tumor progression occurred in 30 of 266 lung tumors (11%); larger tumors showed a higher incidence of progression. No procedure-related mortality was reported.

**Conclusions:**

Lung RFA can be a valuable option for treating lung metastases in patients with musculoskeletal sarcoma.

## Introduction

The lungs are the most common metastatic site for bone and soft-tissue sarcomas (STS), with lung metastasis occurring in 20–50% of patients with high-grade sarcomas [1–4]. Metastasectomy is the standard treatment to prolong survival in patients with lung metastasis [1–6]. The 5-year survival rate following lung metastasectomy is 15–50% in patients with bone sarcoma and STS. Notably, patients who achieve complete treatment for initial metastasis have better post-metastatic survival than those with incomplete treatment [2, 5, 6]. Nevertheless, even after an apparent complete resection of sarcomatous pulmonary metastases, 40–80% of patients experience recurrence of metastatic lesions in the lung [7, 8]. Systemic chemotherapy is another therapeutic option for managing lung metastatic sarcoma; however, these treatments have not significantly improved patient prognosis [9, 10]. Consequently, outcomes for patients with metastatic disease remain poor, with a median overall survival of 12–20 months [10–15].

Lung radiofrequency ablation (RFA) is a relatively safe and effective therapeutic option for treating unresectable non-small cell lung cancer and lung metastases from colorectal cancer [16–21]. We previously reported the short-term results of 20 patients with bone sarcoma and STS who underwent lung RFA [21]. However, long-term outcomes in patients with lung metastases from sarcomas remain unreported. Therefore, this study retrospectively evaluated the clinical utility of lung RFA in 52 patients with bone sarcoma- and STS-derived lung metastases.

## Patients and methods

### Study design

This study was approved by the Institutional Review Board of Mie University Hospital (H2021-072), and written informed consent for lung RFA was obtained from all patients. The study cohort included 29 men and 23 women with a mean age and standard deviation (±SD) of 55 ± 20.8 years (range, 12–87 years) at the time of the initial lung RFA, with a mean follow-up duration of 49.2 ± 59.8 months (range, 3.1–252.8 months). Twenty patients underwent metastasectomy for lung metastasis prior to the initial lung RFA. Curability was classified into complete and incomplete treatments. Complete treatment was defined as achieving a tumor-free status following the initial lung RFA, whereas cases failing to achieve this status were classified as incomplete treatment. The primary endpoint of this study was survival following the initial lung RFA. The secondary endpoints included the safety of lung RFA and the rate of local tumor progression after lung RFA.

This study included consecutive patients who underwent initial lung RFA for lung metastases from sarcomas between 2003 and 2022. Indications for local treatment of lung metastases and the selection of treatment modalities were determined by orthopedic surgeons, thoracic surgeons, and interventional radiologists based on tumor number and location, pulmonary function, and expected patient prognosis. RFA was selected when surgical resection of lung metastases posed a high risk of significantly diminishing pulmonary function or when the patient declined surgery. Patients with a maximum tumor diameter >4.0 cm, abnormal coagulability (including a platelet count <50,000/mL or international normalized ratio >1.5), refractory pleural effusion, and the presence of other uncontrollable malignancies were excluded. The presence of interstitial pneumonia or prior radiation exposure to the lung was also considered an exclusion criterion for lung RFA. Beyond these conditions, lung RFA was offered to patients for whom the procedure was expected to provide a potential cure or extend survival. Among the 52 patients, 49 were intended for complete ablation at the initial RFA, whereas the remaining 3 were planned for incomplete ablation from the outset.

Among the 52 patients with 209 sessions, 18 patients with 59 sessions were included in a previous study that reported short-term results of lung RFA in patients with sarcoma, with a mean follow-up period of 18 months [21].

### RFA procedure

Patients were admitted to the hospital for lung RFA, which was performed under moderate sedation and local anesthesia. Fentanyl citrate (Fentanest; Janssen-Kyowa, Tokyo, Japan) was administered at a dose of 0.1–0.2 mg for analgesia, while lidocaine (Xylocaine®; Astellas Pharma Inc., Tokyo, Japan) was used for local anesthesia. Prophylactic antibiotics (Cefazolin®, Cefamezin®; Astellas) were administered before the procedure and for 1–2 days post-RFA.

Four experienced interventional radiologists conducted RFA, using real-time computed tomography (CT) fluoroscopy (X-Vigor or Aquilion; Canon Medical Systems Corporation, Otawara, Japan) to place the RF electrode in the tumors. An internally cooled electrode (Cool-Tip RF Ablation System; Radionics, Burlington, MA, USA, or Medtronic, Minneapolis, MN, USA) was utilized. The electrode was placed at the center of tumors sized ≤2 cm. For tumors >2 cm, the electrode was positioned sequentially at 2–4 different sites within the tumor, depending on its size and shape. After connecting the electrode to the generator, RF energy was applied for 10–12 min at each tumor site using an impedance-control algorithm.

A maximum of three lung tumors were treated on the same day. The procedural endpoint was immediate coverage of the tumor by ground-glass opacity. Confirmation of this endpoint defined the technical success of the procedure. Any remaining tumors were treated with RFA the following week.

### Follow-up and assessments

Technical efficacy was defined as the coverage of the treated lung tumor by the ablative zone on CT images obtained 2–5 days post-RFA. Two radiologists (T. Y. and M. F.) and three orthopedic surgeons (T. N., K. A., and T. H.) assessed the efficacy of RFA and local tumor progression using follow-up CT images. Complications were evaluated using the Clavien–Dindo classification [22]. Classes III–V were considered major complications. The follow-up protocol included routine physical examinations and CT studies every 3–4 months for the first 2 years, followed by evaluations every 6 months for years 3 and 5. The development of tumor lesions at the edge of the ablated zone on follow-up CT images at least 6 months post-lung RFA was defined as local tumor progression [22].

### Statistical analysis

Patient background characteristics, such as age and tumor size, were reported as means (±SD). Statistical associations between clinicopathological variables were evaluated using chi-square test for qualitative data, and Mann–Whitney *U* test and Wilcoxon signed-rank test for quantitative data. Disease-free interval (DFI) was defined as the time from primary tumor resection to the date of the initial lung metastasis diagnosis.

Survival after the initial RF ablation was defined as the time from the initial RFA for lung metastasis to the date of death or last clinical visit. Survival curves were constructed using the Kaplan–Meier method. Univariate analysis was conducted using log-rank test and Cox proportional hazards model, while multivariate analysis was also conducted using Cox proportional hazards model to evaluate factors associated with survival. Significant factors (*p* values <0.05) identified in the univariate analysis were included as variables in the multivariate analysis. Statistical analysis was performed using the EZR graphical user interface (Saitama Medical Center, Jichi Medical University, Saitama, Japan) for R (R Foundation for Statistical Computing, Vienna, Austria), a modified version of R Commander designed to add statistical functions frequently used in biostatistics. *p* values <0.05 were considered statistically significant.

## Results

### Patients’ demographics at initial treatment for lung metastasis

A total of 52 patients were enrolled in this study, which included 14 cases of bone sarcomas and 38 STSs (Table 1). The primary bone sarcomas were histologically classified as follows: 77 osteosarcomas, 3 chondrosarcomas, 2 undifferentiated pleomorphic sarcomas (UPS) of the bone, 1 Ewing’s sarcoma, and 1 chordoma. The primary STSs were classified histologically as follows: 14 leiomyosarcomas, 5 UPSs, 5 synovial sarcomas, and 3 extraskeletal chondrosarcomas, among others. Twelve patients developed metastases at initial presentation, and chemotherapy was administered to 18 patients before the initial RFA.

**Table 1:** Patient’s background

Variables		All (*n* = 52)	
Age	Mean	55 years	
	Range	12 to 87	
Sex	Men	29	
	Women	23	
Tumor	Bone	14	
	Soft tissue	38	
Metastasis at initial presentation	Yes	12	
	No	40	
Disease-free interval	Mean	20 months	
	Range	0–107	
Extra-pulmonary metastasis before lung metastasis	Yes	1	
	No	51	
Chemotherapy before initial RFA	Yes	18	
	No	34	
Number of metastasis at initial RFA	Mean	5	
	Range	1–20	
Maximum diameter of lung metastasis at initial treatment	Mean	13 mm	
	Range	3–40 mm	
Metastasectomy before initial RFA	Yes	20	
	No	32	

*RFA* Radiofrequency ablation

The mean and median maximum sizes of lung metastases at the initial lung RFA were 13.3 (± 8.1) mm and 10 mm (range, 3–40 mm), respectively, while the mean and median numbers of metastases were 5 (±4.0) and 2 (range, 1–20), respectively. Twenty-seven patients achieved complete treatment. Age (*p* = 0.0215, Mann–Whitney *U* test) and the number of metastases (*p* < 0.0001, Mann–Whitney *U* test) were associated with curability (Table 2).

**Table 2:** Patient’s background separately for curability

Variables		Complete ablation (*n* = 27)	Incomplete ablation (*n* = 25)	*p* value
Age	Mean	48 years	61 years	0.0215
	Range	19–82	12–87	
Sex	Men	14	15	0.588
	Women	13	10	
Tumor	Bone	7	7	1
	Soft tissue	20	18	
Metastasis at initial presentation	Yes	6	6	1
	No	21	19	
Disease-free interval	Mean	28 months	12 months	0.144
	Range	0–107	0–68	
Extra-pulmonary metastasis before lung metastasis	Yes	1	0	1
	No	26	25	
Chemotherapy before initial RFA	Yes	16	16	0.781
	No	11	9	
Number of metastasis at initial RFA	Mean	2	6	<0.0001
	Range	1–7	1–17	
Maximum diameter of lung metastasis at initial treatment	Mean	12 mm	14 mm	0.485
	Range	3 to 35	5 to 33	
Metastasectomy before initial RFA	Yes	12	8	0.404
	No	15	17	

*RFA* Radiofrequency ablation

## Survival after initial RFA for lung metastasis

**Fig. 1:**
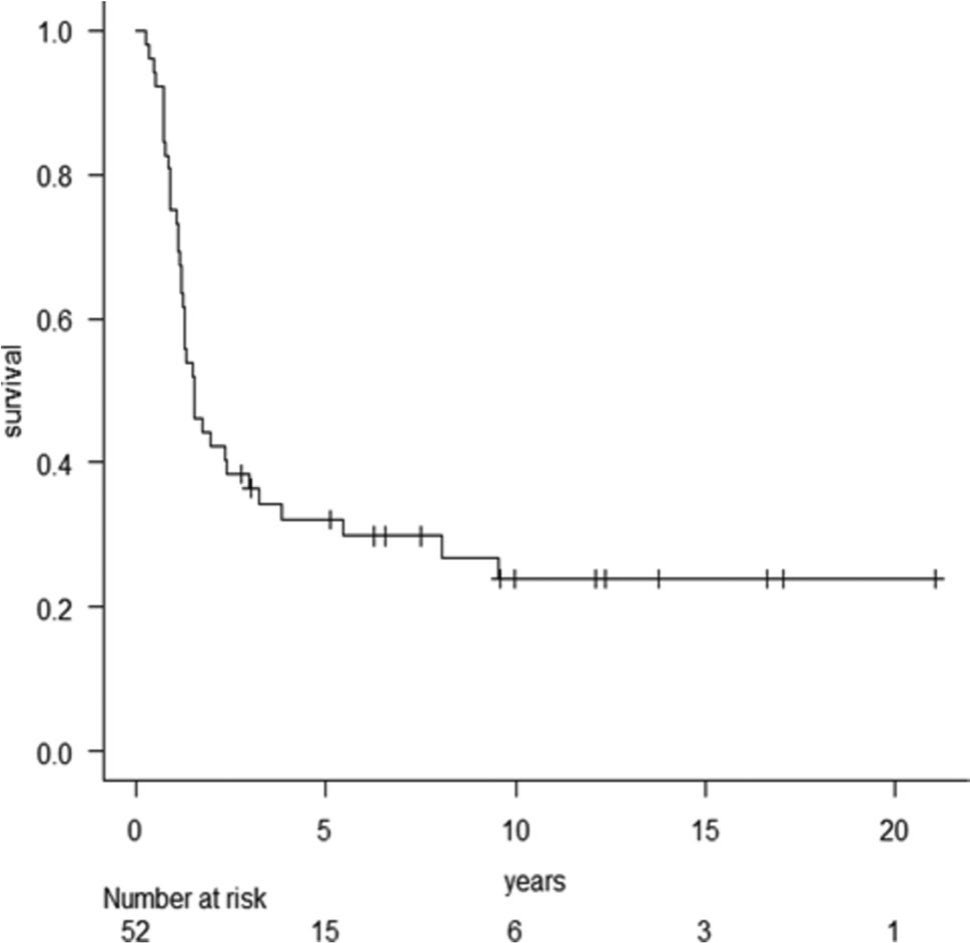
Kaplan–Meier curves illustrating the survival of 52 patients following initial RFA

**Fig. 2:**
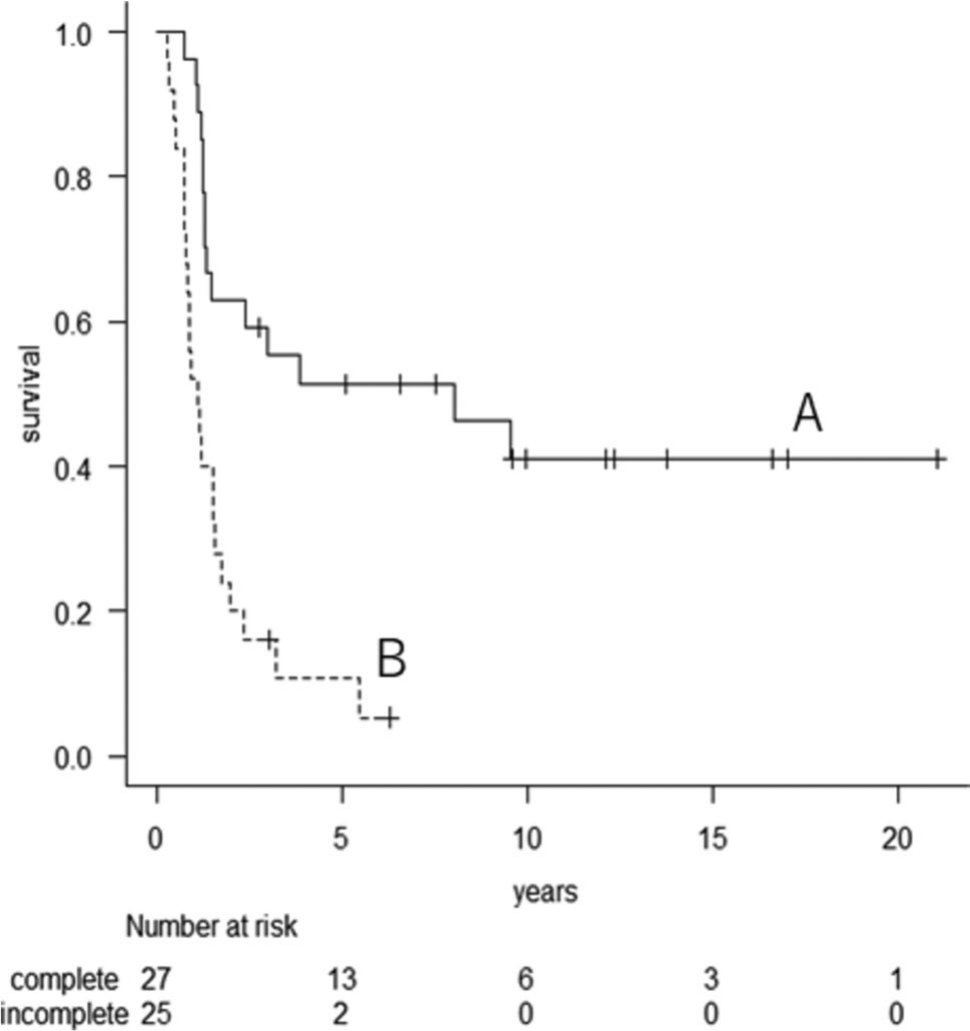
Kaplan–Meier curves depicting survival based on the curability of the initial RFA (**A**: complete treatment, **B**: incomplete treatment)

At the final follow-up, 14 patients remained alive, while 38 had died from the disease; 11 patients showed no evidence of disease. Among the 38 deceased patients, 36 died due to the progression of lung metastases, one from adrenal metastasis, and another from brain metastasis. The 3- and 5-year survival rates were 36.4% (95% confidence interval [CI] = 23.7–49.3%) and 32.2% (95% CI = 20–45.0%; Fig. 1), respectively. The median survival time was 18.4 months. Univariate analysis revealed that DFI (*p* = 0.013) and curability (*p* = 0.0002) were significant predictors of survival after initial RFA (Table 3). Multivariate analysis confirmed that curability and DFI remained significant. The median survival time for the 27 patients with complete treatment was 96.7 months, compared to 13.1 months for the 25 patients with incomplete treatment (*p* < 0.0001, log-rank test). The 3- and 5-year survival rates after initial RFA for the 27 patients with complete treatment were 55.3% (95% CI = 34.9–71.7) and 51.4% (95% CI = 31.3–68.2), respectively, while the rates for the 25 patients with incomplete treatment were 16% (95% CI = 5.0–32.5%) and 10.7% (95% CI = 2.2–27%), respectively (Fig. 2).

**Table 3:** Prognostic variables for predicting survival after initial RFA

Variables		Univariate analysis			Multivariate analysis		
		HR	95% CI	*p* value	HR	95% CI	*p* value
Age	Years	1.011	0.9944–1.027	0.203			
Sex	Women	1					
	Men	1.191	0.6248–2.271	0.595			
Tumor	Bone	1					
	Soft tissue	1.367	0.6247–2.992	0.434			
Metastasis at initial presentation	No	1					
	Yes	0.867	0.3969–1.895	0.721			
Metastasectomy before RFA	No	1					
	Yes	0.8394	0.443–1.59	0.591			
Maximum diameter of metastasis	mm	1.024	0.9848–1.066	0.231			
Number of metastasis		1.054	0.9872–1.126	0.115			
Curability of initial	Complete	1			1		
RFA	Incomplete	3.714	1.851–7.451	0.0002	3.217	1.603–6.456	0.00101
Chemotherapy	No	1					
	Yes	1.325	0.6773–2.593	0.411			
DFI	Month	0.7989	0.9626–0.9955	0.013	0.981	0.9639–0.9984	0.0325

*RFA* Radiofrequency ablation, *DFI* Disease-free interval, *HR* Hazard ratio, *CI* Confidence interval

**Fig. 3:**
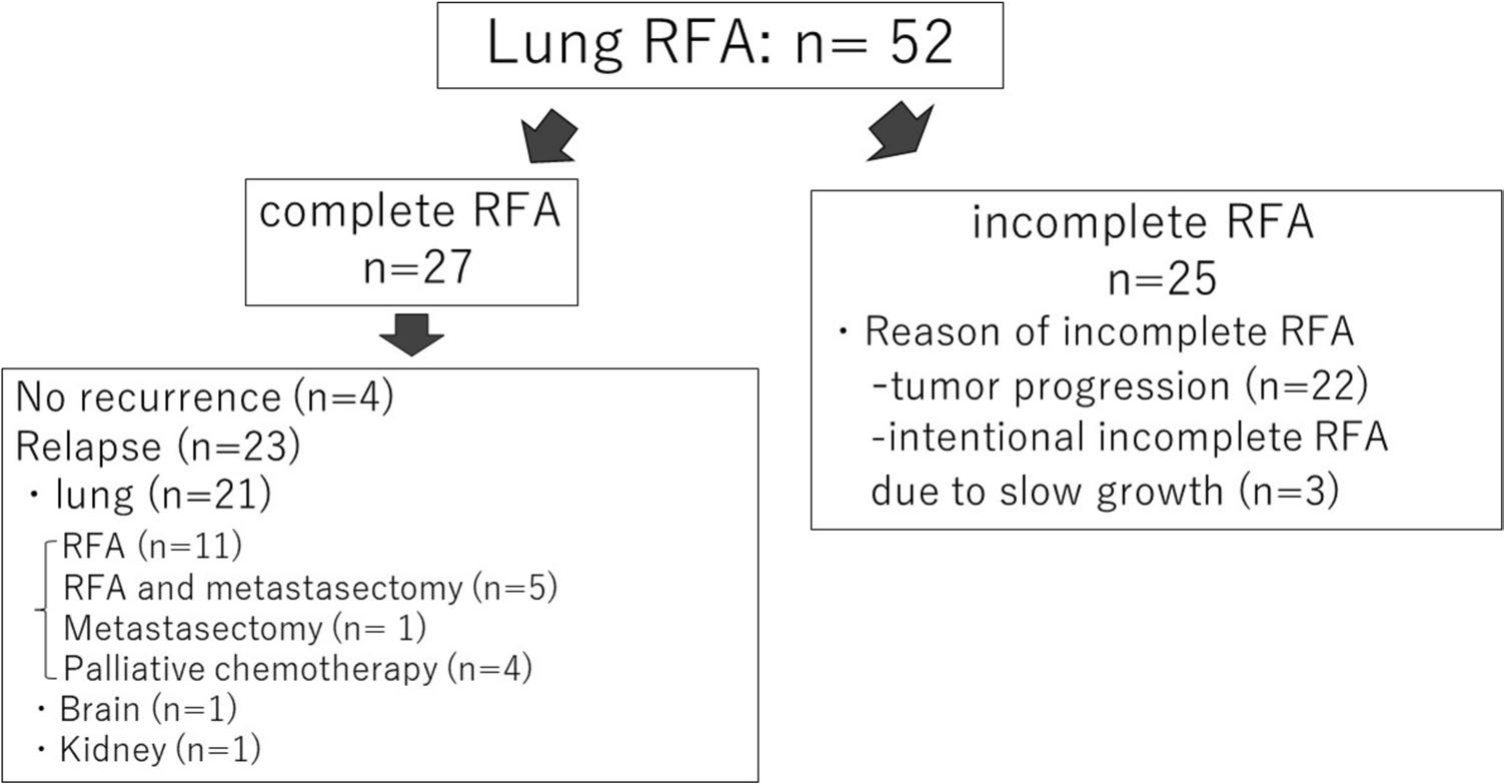
Flowchart outlining the treatment for lung metastasis in 52 patients

Of the 27 patients who achieved complete treatment at the initial RFA, four had no new lung metastases. In contrast, 23 patients developed new metastases and/or local relapse. Among these 23 patients, 21 developed lung metastases. Eleven patients underwent additional lung RFA, five received both lung RFA and metastasectomy, and one underwent metastasectomy alone. One patient developed a brain metastasis, and another developed kidney metastases (Fig. 3). Thirteen patients survived for >5 years after the initial lung RFA.

Of the 25 patients with incomplete treatment, 2 survived for >5 years. One patient had extraskeletal foot chondrosarcoma, and during the initial RFA, six lung metastases were observed. Although the metastases grew slowly, two tumors were treated with RFA due to an increase in size. The patient died of metastasis 65 months after the initial RFA. The other patient was an 88-year-old woman with leiomyosarcoma who had 10 lung metastases. They received systemic chemotherapy; however, one metastasis rapidly grew to 33 mm, prompting RFA and radiotherapy. At the final follow-up, 75 months after the initial RFA, the patient showed no evidence of disease due to a complete response to chemotherapy for the remaining metastases. Of the 25 patients with incomplete treatment, 22 did not achieve complete treatment owing to rapid disease progression during the interval between multiple planned RFA sessions. Nevertheless, 16 of the 22 patients received a combination of systemic chemotherapy and RFA. The remaining three patients (two with extraskeletal chondrosarcoma and one with chordoma) had multiple slow-growing metastases, and incomplete RFA was intentionally performed (Fig. 3).

## Local tumor control and complications of RFA

### Local tumor control

In total, 324 lung metastases were treated across 209 RFA sessions. The technical success rate was 100% (209/209). However, one tumor was not completely ablated on follow-up CT images, necessitating additional RFA the following week. Thus, the technical efficacy rate was 99.5% (208/209). Of the 342 tumors, 266 (78%) were followed using serial CT images for >6 months. Local tumor progression occurred in 30 of the 266 tumors (11%), with larger tumors being more likely to progress (*p* < 0.0001, Mann–Whitney *U* test). Among the 28 tumors ≥2 cm, local progression was found in 10 tumors (36%), whereas progression occurred in 20 of the 235 tumors <2 cm (8%) (*p* = 0.0002, chi-square test).

### Complications

Pneumothorax developed in 137 of 209 sessions (64%), with chest drainage required in 75 of these sessions (54.7%). Other complications included pleural effusion (*n* = 7), hemothorax (*n* = 3), pleuritis (*n* = 1), bronchopleural fistula (*n* = 1), temporary phrenic nerve palsy (*n* = 1), and diaphragmatic hernia (*n* = 1). No procedure-related deaths were reported following lung RFA. According to the Clavien–Dindo classification [23], major complications included: class IIIa in 76 sessions (drainage for pneumothorax and hemothorax), class IIIb in one session (laparoscopic repair for diaphragmatic hernia), and class IVb in one session (septic shock due to bronchopleural fistula).

No patients developed respiratory symptoms following lung RFA sessions.

## Discussion

In the present study involving 52 patients with bone sarcoma and STS who presented with lung metastasis following initial lung RFA, the 3- and 5-year survival rates were 36.4% and 32.2%, respectively, with a median survival time of 18.4 months. Among the 27 patients who underwent complete treatment after the initial lung RFA, the median survival time was 96.7 months, compared to 13.1 months for the 25 patients with incomplete treatment. Local tumor progression after RFA was observed in 30 of 266 tumors (11%), likely occurring in larger tumors. No deaths related to the lung RFA procedure were reported; however, there were 78 major complications according to the Clavien–Dindo classification. Of these complications, chest drainage under local anesthesia was required for 76 patients due to pneumothorax or hemothorax.

Although the treatment approach for advanced stages differs between bone sarcoma and STS, the prognosis for recurrent disease is similar and remains poor, with a 5-year post-relapse survival rate of less than 20% [9–15]. Surgical resection of lung metastases is the standard therapeutic option to prolong patient survival [1–6]. However, due to the high incidence of surgical mortality and morbidity, repeat metastasectomy is often performed reluctantly [24, 25]. In this context, RFA has been utilized as a local treatment for both primary and metastatic lung tumors in patients deemed unsuitable for surgery [16–21].

The advantages of RFA include its minimal invasiveness and repeatability, with several studies on lung RFA for various cancer types reporting favorable outcomes. However, while RFA results have been documented in patients with sarcomas, including bone, soft tissue, and viscera [21, 26–28], studies specifically involving patients with bone sarcoma and STS are limited [21, 26]. For example, Koelblinger et al. reported clinical outcomes after RFA in 22 patients with 55 completely treated lung metastases [26]. The mean survival time was 51 months, with 2- and 3-year survival rates of 94% and 85%, respectively, and a median follow-up period of 20 months. In a larger cohort, Sato et al. evaluated lung RFA in 46 patients with 144 tumors, including 19 with bone sarcoma and STS [27]. The mean and median follow-up periods were 23.9 and 16.7 months, respectively. The 3-year survival rate after the initial RFA for 15 patients with complete treatment was 63.5%, whereas the rate was 37.7% for 31 patients with incomplete treatment. Similarly, Palussière et al. described the clinical outcomes of RFA in 29 patients, including 18 with bone sarcoma and STS [28]. The 1- and 3-year survival rates were 92.2% and 65.2%, respectively. In line with these reports, our study found a 3-year survival rate of 55.3% among the 27 patients who achieved complete treatment following initial RFA. Notably, 23 of these patients (85%) developed new lung metastases and/or local relapses, indicating that repeat RFA should be considered for ongoing tumor control during follow-up.

Consistent with existing guidelines, the number of tumors was a significant factor in achieving complete treatment. Guidelines often recommend resection of limited metastases, particularly when there are up to two or three lung metastases, to enhance long-term survival as fewer tumors are associated with a higher likelihood of complete tumor ablation [29]. Notably, although the role of RFA in patients with incomplete ablation remains unclear, repeat lung RFA can help reduce total tumor volume in such patients, especially if the metastasis is slow-growing or responsive to chemotherapy. In support of this fact, two patients in this study who did not achieve complete treatment after initial RFA survived for >5 years post-RFA due to slow-growing or chemosensitive metastases.

Age was not found to be a prognostic variable for predicting survival although it was related to the achievement of complete RFA. One possible reason for this association is that sarcoma treatment tends to be less aggressive in older patients than in younger ones due to concerns about complications, and in some cases, patients decline metastasectomy. In this study, complete RFA was a reasonable therapeutic option with acceptable survival rates in older patients. However, the indications for RFA should be discussed by multidisciplinary teams. Patients with favorable American Society of Anesthesiologists Physical Status scores may benefit from treatment regardless of age [30]. Regarding tumor control, De Baère et al. indicated that of 1,037 treated metastases, 86 exhibited local progression, with local tumor progression rates of 5.9, 8.5, 10.2, and 11.0% at 1, 2, 3, and 4 years, respectively [31]. Other reports have shown that lung RFA for lesions <2 cm yielded local control rates exceeding 90% [32, 33]. Thus, tumor size can be a predictor of local tumor progression. In the present study, 36% of patients with tumors >2 cm experienced local tumor progression, a significantly higher rate compared to the 8% progression rate in patients with tumors <2 cm.

Although RFA is generally considered a safe procedure, various complications can arise [34, 35]. Pneumothorax is a common complication, with drainage requirements varying between 5 and 50%. In this study, chest drainage was performed after 36% (75/209) of the lung RFA sessions. Although the proportion of patients with pneumothorax requiring drainage was relatively high, this may reflect our emphasis on preventing major lung collapse and ensuring patient safety. Furthermore, according to the Clavien–Dindo classification, two sessions resulted in major complications in addition to chest drainage under local anesthesia.

This study has several limitations. First, it was a retrospective analysis, and we did not compare the outcomes of metastasectomies with those of radiotherapy. Additionally, confirmatory biopsies were not routinely performed. The heterogeneity of the cohort (reflecting diverse histologic types with biological behavior, a broad age range, and various concomitant therapies) confounds the interpretation of the study findings. Furthermore, our cohort was relatively small due to the rarity of sarcoma even though it represents the largest series of lung RFA for musculoskeletal sarcoma metastasis. A selection bias regarding tumor aggressiveness clearly existed between the two groups. Although this study showed complete ablation as an independent survival factor, the extent to which complete ablation contributed to longer survival cannot be firmly determined in this retrospective study. Nevertheless, our results may aid in determining the indications for RFA in patients with bone metastases and STS.

In conclusion, lung RFA may be a valuable option for treating lung metastases in patients with musculoskeletal sarcomas.

## Data Availability

The data that support the findings of this study are available from the corresponding author upon reasonable request.
